# Epidemiological characteristics and genomic analysis of respiratory adenovirus in Lanzhou City from 2023 to 2025

**DOI:** 10.3389/fcimb.2025.1680735

**Published:** 2026-01-07

**Authors:** Biao Wang, Shu Liang, Huan Wei, Miao Wang, Xiaoshu Zhang, Maoxing Dong, Hui Zhang

**Affiliations:** 1 Lab for Viruses, Gansu Provincial Center for Disease Control and Prevention, Lanzhou, China; 2 Public Health School, Gansu University of Chinese Medicine, Lanzhou, China

**Keywords:** respiratory infections, adenovirus, epidemiological characteristics, genetic features, phylogenetic trees

## Abstract

**Objective:**

Human adenoviruses (HAdVs) are common pathogens causing inflammation in the upper respiratory tract, lungs, conjunctiva, urinary tract, and gastrointestinal tract. They can lead to severe, even fatal infections in immunocompromised patients, representing a significant public health threat. To comprehensively assess the impact of HAdV on public health, this study investigated the epidemiological and genetic characteristics of respiratory adenoviruses in Lanzhou City from 2023 to 2025.

**Methods:**

From 2023 to 2025, 1,269 acute respiratory infection specimens were collected from sentinel hospitals in Lanzhou City. HAdV nucleic acids were detected using real-time fluorescent quantitative PCR (qPCR). A portion of HAdV-positive specimens underwent viral isolation using Hep-2 cells. Successfully isolated adenovirus strains were subjected to whole-genome sequencing. Phylogenetic trees were constructed for the HAdV whole genome, Penton base, Hexon, and Fiber genes, and protein variant sites were analyzed.

**Results:**

The overall HAdV positivity rate in acute respiratory infection specimens from Lanzhou City was 7.88% (100/1269) from 2023-2025, with a higher positivity rate observed in children aged ≤15 years. HAdV prevalence displayed seasonal variations, with significantly higher positivity rates in autumn and winter (10.83%, 73/674) compared to spring and summer (4.54%, 27/595). Whole-genome sequencing identified the predominant circulating HAdV strain in Lanzhou City during the study period as HAdV-B, all of which were HAdV-3. Phylogenetic analyses of the Penton base, Hexon, and Fiber genes revealed high genetic homology between all nine Lanzhou HAdV-B strains and the Guangzhou candidate vaccine strain (GenBank: DQ099432.4), clustering on the same branch. Furthermore, the Penton base RGD domain and Hexon antigenic epitope of the nine Lanzhou HAdV-B strains remained highly conserved compared to the Guangzhou candidate vaccine strain, showing no significant mutations.

**Conclusion:**

The prevalent HAdV strain in Lanzhou City from 2023 to 2025 was type B3. Based on these findings, it is tentatively suggested that the vaccine candidate strain from Guangzhou may provide good immune protection against these prevalent strains, though verification through clinical trials is required.

## 1 Introduction

Acute respiratory infections (ARI) are a major cause of high morbidity and mortality rates in children and infants worldwide. Statistics show that approximately 80% of acute respiratory infections are caused by viruses (Choi et al., 2018), with HAdV accounting for approximately 2%~15% of respiratory infections in children (Finianos et al., 2016; Jain et al., 2015). HAdV belongs to the genus Human Adenovirus of the family Adenoviridae. It is a non-enveloped linear double-stranded DNA virus with an icosahedral symmetrical structure (Rowe et al., 1953). Its genome size ranges from 34 to 37 kb and can encode over 40 proteins (Lion, 2014; Davison et al., 2003). The HAdV capsid consists of three major components: Penton base protein, Hexon protein, and Fiber protein (Russell, 2009; van Oostrum and Burnett, 1985). Among these, the arginine-glycine-aspartic acid (RGD) motif of the Penton protein interacts with host cell integrins, a critical step in promoting viral endocytosis (Zubieta et al., 2006). The Hexon protein, a type- and species-specific antigen, is susceptible to immune selection pressure (Feng et al., 2018; Qiu et al., 2012; Yuan et al., 2009). The Fiber protein contains viral receptor-binding sites and plays a crucial role in viral attachment and invasion (Liebermann et al., 1998).

HAdV can infect the upper and lower respiratory tracts of children, causing various respiratory diseases, including bronchitis, laryngitis, tonsillitis, and pneumonia (Louie et al., 2008; Otto et al., 2021). As of March 2024 (data source: http://HAdVwg.gmu.edu/), HAdVs have been classified into seven subgenera (A–G) based on their biological characteristics and genetic features, with 116 serotypes identified to date. Previous studies have shown that different HAdV serotypes exhibit varying tissue tropisms, leading to distinct organ-specific diseases, such as respiratory infections (subtypes B, C), conjunctivitis (subtypes B, D, E), gastroenteritis (subtypes F, G), meningitis (subtypes B, C), and cystitis (subtypes A, B) (Ismail et al., 2018; Hierholzer et al., 1974; Lin and Chen, 2019; Radke and Cook, 2018; Song et al., 2012). Currently, only the United States has a bivalent HAdV-4 and HAdV-7 vaccine for military personnel (Collins et al., 2020). Although no vaccine targeting respiratory adenoviruses has been approved for use in China, efforts to develop such a vaccine are underway. For example, a research team from Guangzhou Medical University successfully developed a trivalent candidate vaccine (containing HAdV-3, HAdV-7, and HAdV-55), which induced effective antibodies against these three viruses in mice (Liu et al., 2018). However, current treatment for HAdV infections primarily relies on symptomatic supportive therapy, with no specific antiviral drugs available, which limits the effective prevention and treatment of HAdV infections (Dodge et al., 2021).

Gansu Province, located in the northwestern inland region of China, is characterized by complex and diverse climatic conditions. Concurrently, Lanzhou City, a critical corridor and logistics hub within the “Belt and Road” initiative, has seen its geographical advantages and nodal functions become increasingly prominent. While previous studies have documented the epidemiological characteristics of acute respiratory infection pathogens in this area (Wang et al., 2025), this study aimed to provide a more in-depth understanding of the epidemiological and genomic features of HAdV in Lanzhou City. By analyzing clinical samples collected from a sentinel hospital in Lanzhou, Gansu Province, between 2023 and 2025, this study aims to elucidate the prevalence patterns and molecular characteristics of this pathogen, not only contributes to a deeper understanding of the epidemiological characteristics and evolutionary patterns of HAdV-B3 but also lays a crucial foundation for developing highly effective vaccines and formulating public health strategies.

## 2 Materials and methods

### 2.1 Sample collection and nucleic acid testing

This study selected cases of acute respiratory infections from a designated tertiary hospital in Lanzhou City, Gansu Province, between 2023 and 2025. Inclusion criteria: ① Symptoms consistent with acute infection (meeting at least one of the following): fever, chills; abnormal white blood cell distribution count (decreased or increased; adults (≥16 years): (4.0–10.0) × 10^9/L; children (6–15 years): (4.5 - 13.5) × 10^9/L; children (1–5 years): (6.0 - 15.5) × 10^9/L); ② clinical symptoms (meeting at least one of the following criteria): rhinorrhea, cough with sputum production, wheezing, pharyngeal or laryngeal edema or pain, chest tightness or pain, fatigue, abdominal pain, or diarrhea. Qualified medical staff at the sentinel hospital strictly followed the monitoring protocol to collect nasopharyngeal swab specimens and gathered case data. Specimens were stored at 4°C within 24 h of collection and transported to the laboratory. Specimens submitted for testing after 24 h must be stored at -70°C. This study employed the Xi’an Tianlong Nucleic Acid Rapid Extraction Kit (magnetic bead method) to extract total viral nucleic acids from the samples. HAdV detection was performed using the Respiratory Pathogen Nucleic Acid Detection Kit (Catalog No.: A773VYH-50T) developed by Beijing Zhuocheng Huisheng Biotechnology Co., Ltd., in conjunction with the ABI Q5 Real-Time Fluorescent Quantitative PCR System. The real-time quantitative PCR reaction system was prepared as follows: 18 µL of Mix-A nucleic acid amplification reaction buffer, 2 µL of enzyme mixture, and 5 µL of nucleic acid from the kit. The reaction conditions were as follows: 50°C for 10 min, 95°C for 30 s, 95°C for 5 s, and 60°C for 30 s, repeated for 45 cycles. The detection channel was set to VIC. A Ct value ≤38 with a distinct S-shaped amplification curve was considered HAdV-positive. The minimum detection limit of the kit was 500 copies/mL. All experimental steps were strictly followed according to the manufacturer’s instructions.

### 2.2 Cell culture and virus isolation

In accordance with the virus isolation method outlined in Appendix 9 of the Technical Guidelines for the Prevention and Control of Human Adenovirus Respiratory Infections (2019 Edition) issued by the Chinese Center for Disease Control and Prevention, HAdV-positive throat swab samples identified using qPCR were subjected to isolation and culture.This study used well-grown Hep-2 cells and placed them in a 37°C, 5% CO_2_ incubator. The cells were allowed to grow to 85%–90% confluence before use in the experiments. HAdV-positive throat swab samples identified using qPCR were centrifuged at 12,000 rpm for 10 min. Subsequently, 200 µL of the throat swab suspension was inoculated into cell culture tubes containing the aforementioned Hep-2 cell monolayers. The tubes were placed in a 37°C, 5% CO_2_ incubator for culture, and cytopathic effects were observed daily. The affected cells became enlarged and rounded, with gaps appearing between adjacent cells, forming grape-like clusters. Samples were promptly harvested when 76%–100% of the cells exhibited cytopathic effects (CPE). Non-lesioned samples were collected after 5–7 days or after extensive cell detachment. After three freeze-thaw cycles, the samples underwent three blind passages. The third viral isolate was subjected to qPCR analysis. Isolates LZ09350-23, LZ10023-23, LZ10026-23, LZ129046-24, LZ129066-24, LZ129067-24, LZ121645-24, LZ121708-24, and LZ121731-24.

### 2.3 Whole genome sequencing

600µL of each of the nine inactivated cultures, which had been incubated at 56 °C for 30 min and then allowed to stand for 20 min, were sent to Shanghai Bojie Medical Technology Co., Ltd. for second-generation whole-genome sequencing. Quality control was performed on raw sequencing data.

### 2.4 Sequence alignment and analysis

The HAdV genome sequences obtained through sequencing were submitted to the National Center for Biotechnology Information (NCBI) GenBank. Homology searches were performed against the database using the Basic Local Alignment Search Tool (BLAST) to identify the most closely related sequences. Genome sequences of the Chinese Guangzhou vaccine candidate strain (GenBank: DQ099432.4), the US vaccine strain (GenBank: AY594255.1), and a reference strain of HAdV-B were downloaded from GenBank and used as reference sequences for comparison. Nucleotide sequence alignment was performed between the sequenced genome and reference sequences using MEGA 11 software. Subsequently, sequence similarity analysis was conducted using BioEdit v7.0.9 software. Phylogenetic trees for the complete genome, Penton base gene, Hexon gene, and Fiber gene were constructed using the Maximum Likelihood (ML) method in MEGA 11 software, with bootstrap analysis performed with 1000 replicates. Furthermore, sequence similarity analyses of the HAdV genes and their encoded proteins were conducted using MEGA 11 and BioEdit v7.0.9 software. Detailed analyses of amino acid variation sites in the Penton base protein, Hexon protein, and Fiber protein were performed using DNAMAN9.0 software.

### 2.5 Statistical analysis

Data analysis and statistical comparisons were performed using IBM SPSS Statistics version 26.0. Data are presented as numbers and percentages. Comparisons of continuous variables with normal distributions were performed using t-tests, and non-normally distributed variables were analyzed using the Mann-Whitney U test. Categorical data were analyzed using the χ² or Fisher’s exact tests. The 95% confidence intervals (CI) for positive rates were calculated using R version 4.5.1. Statistical significance was set at P < 0.05.

## 3 Results

### 3.1 Epidemiological characteristics of HAdV

**Figure 1:**
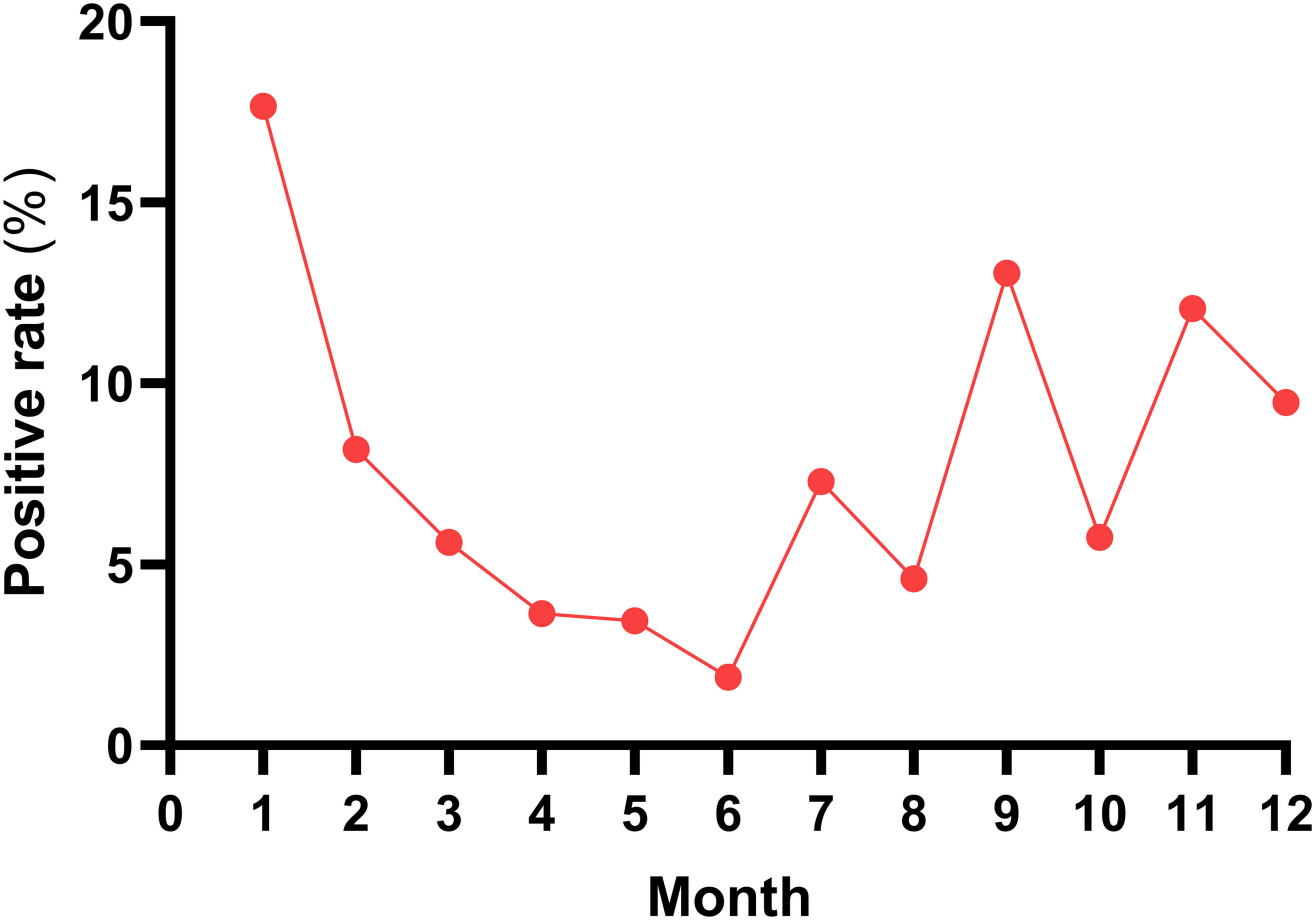
Monthly positive rate trend of HAdV in Lanzhou City, 2023–2025.

Between 2023 and 2025, 1,269 cases of acute respiratory infections (ARI) were collected, with a positive detection rate of 7.88% (100/1,269) for HAdV. Among different genders, there were 730 male cases (730/1269, 57.53%) and 539 female cases (539/1269, 42.47%), with a male-to-female ratio of approximately 1.35:1. The HAdV positivity rates in the male and female groups were 8.08% (59/730) and 7.61% (41/539), respectively, with no statistically significant difference between the two groups (χ² = 0.756, P = 0.097). When grouped by age, the HAdV positivity rates for each age group were as follows: ≤1 year old group 5.03% (8/159), 1–5 years old group 8.70% (44/506), 5–15 years old group 9.83% (46/468), and >60 years old group 5.26% (2/38); However, no HAdV was detected in the 15–25 years old group (35 cases) and the 25–60 years old group (63 cases). The difference in HAdV positivity rates between the ≤15 years old group and the>15 years old group was statistically significant (χ² = 7.660, P < 0.05). The distribution of HAdV-positive detection rates by season was as follows: spring 4.18% (15/359), summer 5.08% (12/236), autumn 10.29% (28/272), and winter 11.19% (45/402). The difference in HAdV-positive detection rates between autumn and winter and spring and summer was statistically significant (χ² = 17.240, P < 0.001) (**Table 1**). The HAdV positivity rate in Lanzhou showed a downward trend from January to June and an upward trend from June to December. See **Figure 1**.

**Table 1:** HAdV detection results.

Characteristics	Sample number	Positives number	Positives rate (%)	95% CI for the positive rate(%)	*P*-value (χ²)
Age					0.006(7.660)
≤1year	159	8	5.03	2.36-10.01	
1-5years	506	44	8.70	6.46-11.58	
5-15years	468	46	9.83	7.36-12.98	
15-25years	35	0	0	0	
25-60years	63	0	0	0	
>60years	38	2	5.26	0.92-19.07	
Gender					0.756(0.097)
Males	730	59	8.08	6.26-10.36	
Females	539	41	7.61	5.58-10.26	
Season					<0.001(17.240)
Spring	359	15	4.18	2.44-6.95	
Summer	236	12	5.08	2.78-8.94	
Autumn	272	28	10.29	7.07-14.68	
Winter	402	45	11.19	8.36-14.79	
Total	1269	100	7.88	6.49-9.54	

### 3.2 HAdV whole genome sequence analysis and subgenus analysis

Whole-genome sequencing of nine HAdV strains was performed using Illumina Miniseq, yielding complete genome sequences.The average sequencing depths were as follows: Isolate 1 (LZ09350-23) 16,481; Isolate 2 (LZ10023-23) 19,490; Isolate 3 (LZ10026-23): 25,269; Isolate 4 (LZ129046-24): 4,095; Isolate 5 (LZ129066-24): 4,595; Isolate 6 (LZ129067-24): 1439; Isolate 7 (LZ121645-24) 5087; Isolate 8 (LZ121708-24) 4466; Isolate 9 (LZ121731-24) 3726. Q30 values were as follows: Isolate 1 (LZ09350-23) 95.17%; Isolate 2 (LZ10023-23) 95.21%; Isolate 3 (LZ10026-23) 95.02%; Isolate 4 (LZ129046-24) 93.92%; Isolate 5 (LZ129066-24) 94.21%; Isolate 6 (LZ129067-24) 93.86%; Isolate 7 (LZ121645-24) 94.26%; Isolate 8 (LZ121708-24) 94.18%; Isolate 9 (LZ121731-24) 93.90%.After assembly, the genome sizes of these nine strains were: 35,258 bp, 35,219 bp, 35,229 bp, 35,263 bp, 35,256 bp, 35,255 bp, 35,261 bp, 35,260 bp, and 35,254 bp. Their respective G+C contents were: 41.07%; 42.49%; 42.53%; 49.71%; 49.34%; 48.41%; 49.49%; 49.76%; 51.16%, there were five males and four females; the age distribution was as follows: 3 cases in the 1–5-year-old group and 6 cases in the 5–15-year-old group. Among these, 3 cases were from 2023 and 6 from 2024. NCBI BLAST analysis of the complete genome sequences, as well as the penton base, hexon, and fiber gene sequences, showed that all nine HAdV samples were HAdV-B type and belonged to the HAdV-3 genotype.

### 3.3 Similarity analysis

Whole-genome sequencing was performed on the 9 HAdV-B3 strains obtained from Lanzhou City, and the results showed nucleotide sequence similarity ranging from 99.80% ~ 99.90%. When these nine strains were compared with the vaccine candidate strain from Guangzhou, China (Genbank: DQ099432.4), the whole-genome sequence similarity ranged from 99.70% ~ 99.80%. The nucleotide sequence similarity ranges for the penton base, hexon, and fiber genes were 99.20%~99.80%, 99.80%~99.90%, and 99.70%~99.80%, respectively. Further analysis revealed that compared to the US vaccine strain (Genbank: AY594255.1), the whole-genome sequence similarity of the Lanzhou strain ranged from 96.00% ~ 96.10%, the sequence similarity of the penton base gene ranged from 99.00% ~ 99.40%, the sequence similarity of the hexon gene ranged from 95.60% ~ 95.70%, and the sequence similarity of the fiber gene ranged from 62.90% ~ 63.00% (**Table 2**).

**Table 2:** Similarity analysis.

Comparison	Whole genome nucleotide	Penton base		Hexon		Fiber	
		Nucleotide	Amino acid	Nucleotide	Amino acid	Nucleotide	Amino acid
Comparison among 9 HAdV sequences from Lanzhou City	99.80%~99.90%	99.60%~99.90%	99.40%~100%	99.90~100%%	99.80%~100%	99.80%~100%	100%
Comparison among 9 HAdV sequences from Lanzhou City and HAdV-B candidate vaccine strain Guangzhou01	99.70%~99.80%	99.20%~99.80%%	99.20%~99.80%	99.80%~99.90%	99.70%	99.70%~99.80%	99.60%
Comparison among 9 HAdV sequences from Lanzhou City and American HAdV-B HAdV-7 vaccine strain Gomen	96.00%~96.10%	99.00%~99.40%	98.20%~98.60%	95.60%~95.70%	90.30%~90.50%	62.90%~63.00%	30.20%

### 3.4 Phylogenetic analysis

**Figure 2:**
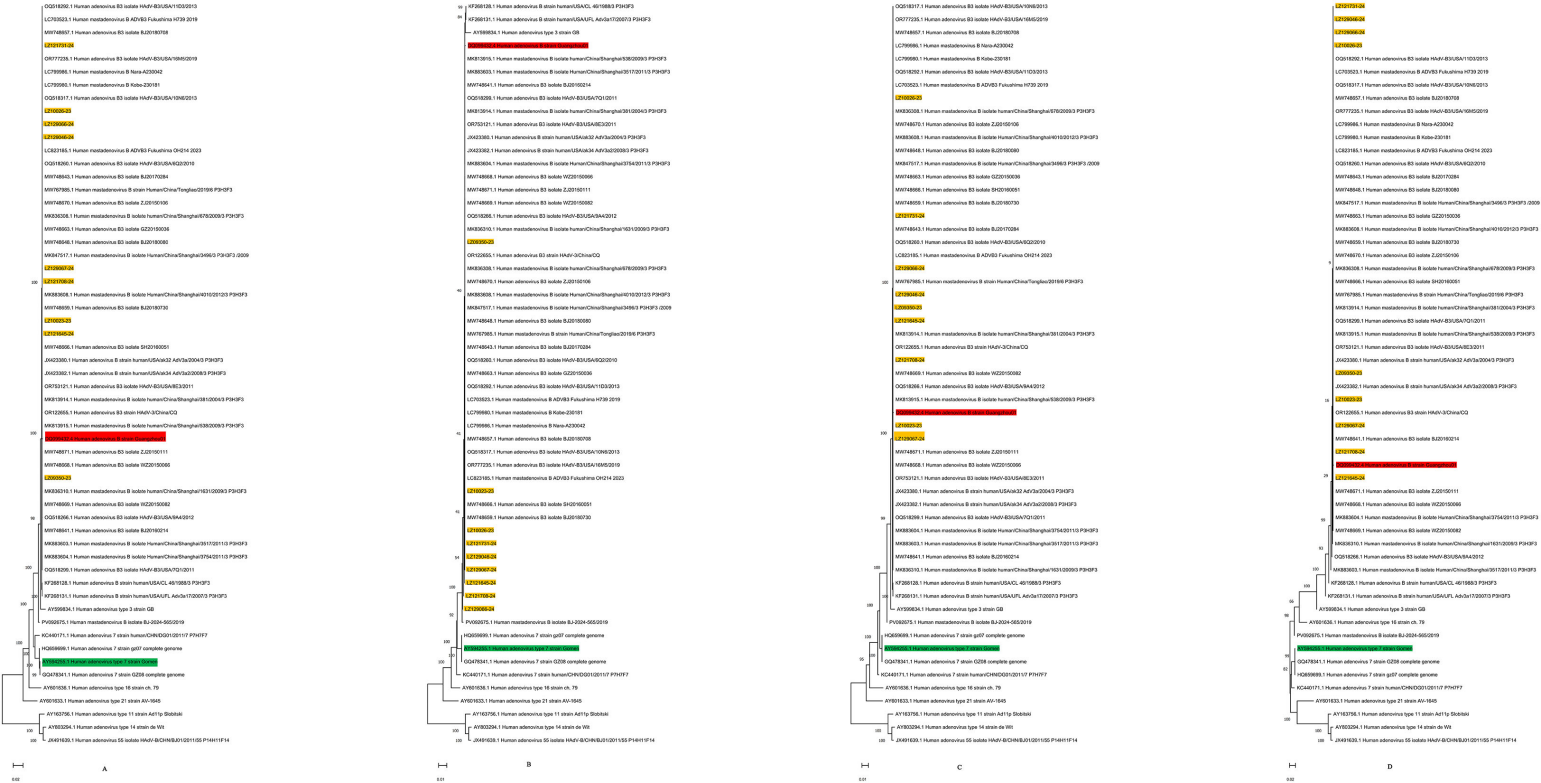
Phylogenetic trees of HAdV-B strains from Lanzhou City based onwhole-genome sequences **(A)**, Penton base gene **(B)**,Hexon gene **(C)**, and Fiber gene **(D)**. The nine HAdV sequences from Lanzhou City are shown in orange. The candidate vaccine strain HAdV-3 from Guangzhou, China (GenBank: DQ099432.4) is marked in red. The HAdV-7 vaccine strain from the United States (GenBank: AY594255.1) is indicated in green.

Based on the full-genome sequences of 9 HAdV-B strains from Lanzhou City obtained in this study, as well as the HAdV-3 Guangzhou vaccine candidate strain (Genbank: DQ099432.4), the US HAdV-7 vaccine strain (Genbank: AY594255.1), and 46 reference strains from the human adenovirus B subgenus, phylogenetic trees were constructed for the HAdV-B whole genome, Penton base gene, Hexon gene, and Fiber gene. Phylogenetic analysis showed that in the HAdV-B whole-genome phylogenetic tree, all 9 HAdV-B strains from Lanzhou clustered with the Chinese HAdV-3 Guangzhou vaccine candidate strain (Genbank: DQ099432.4) on the same branch (**Figure 2A**). Further analysis indicated that in the phylogenetic trees of the Penton base, Hexon, and Fiber genes, the 9 HAdV-B strains from Lanzhou City also clustered with the Chinese HAdV-3 Guangzhou vaccine candidate strain (Genbank: DQ099432.4) in the same branch (**Figures 2B-D**).

### 3.5 Analysis of amino acid variations

Penton base, hexon, and fiber proteins are essential components of the HAdV capsid and serve as important diagnostic targets. The gene regions encoding these three proteins are the most variable in the HAdV genome and are therefore key areas for adenovirus evolution studies (Madisch et al., 2005; Madisch et al., 2007, Miura-Ochiai et al., 2007), playing critical roles in viral infection (Russell, 2009; van Oostrum and Burnett, 1985).Compared to the Chinese HAdV-3 Guangzhou vaccine candidate strain (GenBank: DQ099432.4), the penton base protein of the nine HAdV-B strains isolated in Lanzhou showed one mutation and two insertions, the hexon protein exhibited three amino acid mutations, and the fiber protein displayed a deletion at position S266 in all nine sequences. Detailed results are presented in **Table 3**.

**Table 3:** Analysis of amino acid mutations in 9 HAdV-B strains from Lanzhou City.

Name	Amino acid mutation sites compared to Guangzhou01
Penton base	V14 insert(2),L15insert(2),W189L(9)
Hexon	G256E(8),G256K(1),R832C(9)
Fiber	S266 delete(9)

**Figure 3:**
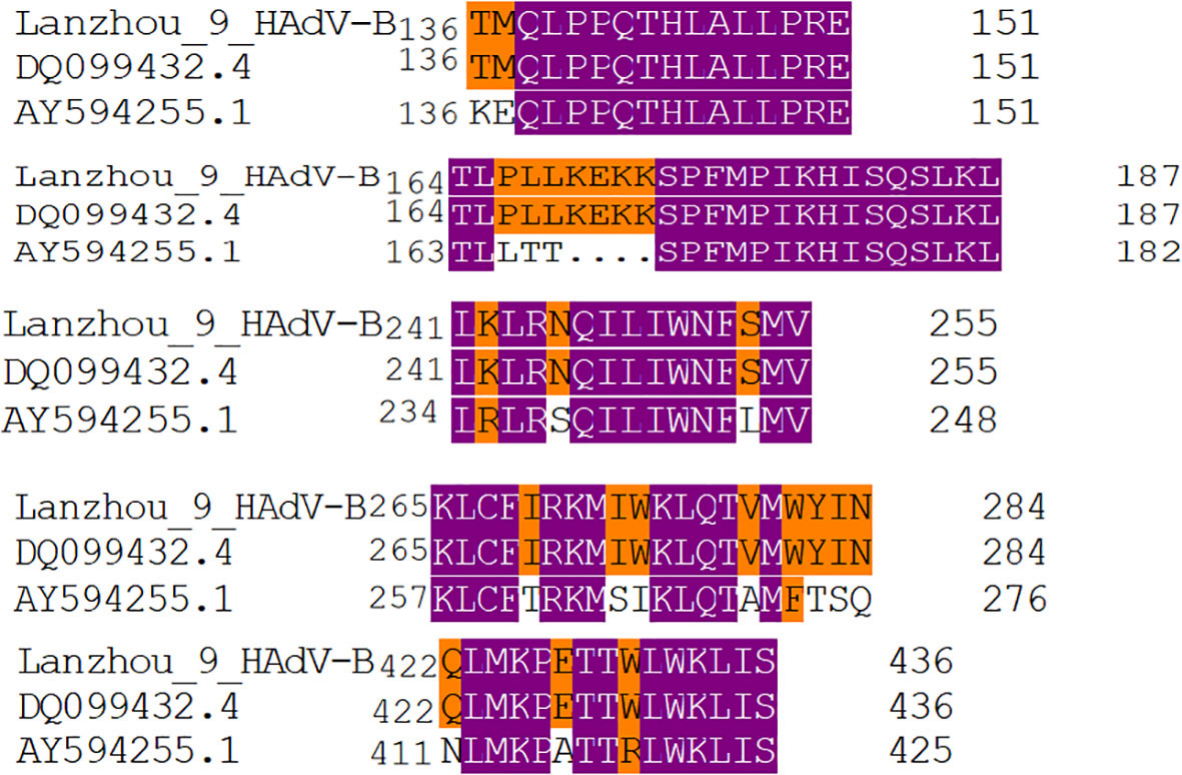
Analysis of serotype-specific neutralizing epitope variations of the HAdV-B hexon protein in Lanzhou City, China: vaccine candidate strain from Guangzhou, China (GenBank: DQ099432.4) and vaccine strain from the United States (GenBank: AY594255.1).

The highly conserved tripeptide sequence Arg-Gly-Asp (RGD) on the pentameric protein binds to integrin αvβ3 or αvβ5 (auxiliary receptors) on the cell surface, promoting viral endocytosis (Shayakhmetov et al., 2005). In this study, the corresponding regions of the 9 HAdV strains circulating in Lanzhou remained unchanged. The Hexon protein region contains serotype-specific neutralizing epitopes that induce the host to produce serotype-specific Ab. The serotype-specific neutralizing epitopes of HAdV-3 Hexon are primarily composed of amino acid fragments 136-151, 164-187, 241-255, 265-284, and 422-437 (Qiu et al., 2012; Yuan et al., 2009). Compared with the Guangzhou vaccine candidate strain (GenBank: DQ099432.4) in China, the Hexon antigenic epitopes of the nine HAdV-B strains from Lanzhou remained unchanged. However, compared with the US vaccine strain (GenBank: AY594255.1), the nine HAdV-B strains from Lanzhou exhibited the following mutations in the Hexon protein serotype-specific neutralizing epitope region: KE136-137TM, LTT165-167PLL, R235K, S238N, L246S, T261I, SI265-266IW, A271V, FTSQ273-276WYIN, N411Q; A416E; R419W (**Figure 3**).

## 4 Discussion

Previous studies have shown that the epidemiological trends of HAdV vary across regions (Cao et al., 2024; Chen et al., 2022). This study analyzed the epidemiological characteristics of HAdV in respiratory specimens from Lanzhou City between 2023 and 2025, with results indicating an HAdV positivity rate of 7.88%. This positivity rate was higher than the 3.42% reported in Jining City, China (Dou et al., 2025), but lower than the 10.86% observed in Suzhou, China (Zang et al., 2025). HAdV is prevalent throughout the year in Lanzhou, with peaks in autumn and winter. This is similar to the findings from Jilin Province, China (Yu et al., 2021), but differs from the summer and winter prevalence patterns observed by Liu et al. (2023).

This study found that the HAdV positivity rate among children under 15 years of age in Lanzhou City was relatively high, with the highest positivity rate observed in the 5–15 age group, reaching 9.83% (46/468), consistent with the findings of a study in Jining City, China (Dou et al., 2025). Additionally, during the later stages of the COVID-19 pandemic, the activity of HAdV-B type viruses (particularly HAdV-B3, HAdV-B7, and HAdV-B21) significantly increased among school-aged children in China (Jin et al., 2025). In contrast, the lowest positivity rate was observed in the 15–60 age group, but positive cases were still detected in the age group over 60 years old. These results suggest that children under 15 years of age in Lanzhou City are a susceptible population for HAdV infection, and scientific prevention and control measures should be strengthened for this age group.

There are significant regional differences in the distribution of HAdV genotypes. Between 2009 and 2020, HAdV outbreaks in China were primarily caused by HAdV-3, HAdV-4, HAdV-7, and HAdV-55 (Liu et al., 2023). The COVID-19 pandemic and implementation of non-pharmaceutical interventions (NPIs) significantly altered the epidemiological characteristics of HAdV, from early 2020 to late 2022, Lanzhou City rigorously implemented multi-tiered NPIs, including city-wide lockdowns, mass nucleic acid testing, social distancing controls, school closures, and mandatory mask mandates in public spaces. While these robust NPIs effectively controlled COVID-19 transmission, they also significantly disrupted the epidemiological patterns of HAdV infection (Wang et al., 2025). The significant decline in HAdV-B3 and HAdV-B7 activity during the pandemic may have weakened herd immunity, particularly among children with limited exposure to the virus, thereby increasing the risk of infection in the post-pandemic period (Huang et al., 2023; Wang et al., 2023; Li et al., 2022). A significant resurgence of HAdV-B3 has been reported in China, the United States, South Korea, and Japan following the COVID-19 pandemic (Jin et al., 2025; Lee et al., 2024; Abdullah et al., 2024; Fukuda et al., 2024). HAdV-B3 is a widely circulating genotype in different regions of China (Wang et al., 2024; Chen et al., 2022). This study identified HAdV-B3 as the circulating strain in the Lanzhou region from 2023 to 2025 using phylogenetic analysis.

The complete genome sequences of all nine HAdV-B strains from Lanzhou City obtained in this study, as well as their Penton base, Hexon, and Fiber gene sequences, were highly similar to those of the Guangzhou vaccine candidate strain (GenBank: DQ099432.4) and clustered together on the same evolutionary branch in the phylogenetic analysis. In contrast, these sequences showed lower similarity to the US vaccine strain (GenBank: AY594255.1). Further comparison revealed that, compared to the Guangzhou vaccine candidate strain (GenBank: DQ099432.4), the Lanzhou HAdV-B strains exhibited certain amino acid mutations in the penton base, Hexon, and Fiber protein regions, including point mutations (W189L, G256E, G256K, R832C) and amino acid insertions (V14 insertion, L15 insertion). Similar amino acid substitutions in the hexon region have been observed in circulating strains from Germany, South Korea, Japan, and Taiwan, China (Haque et al., 2018). The mutation sites identified in this study were not included in previously functionally validated epitopes (Keib et al., 2017; Leen et al., 2004; Chakupurakal et al., 2013; Duan et al., 2021). However, we can not entirely rule out the potential impact of these mutations on antigenicity, which requires further immunological investigations. Given that the Penton base of HAdV is closely associated with viral entry into cells (Shayakhmetov et al., 2005), this study reports the first observation of a V14/L15 insertion in the penton base. The effects of this insertion on the structural stability and other properties of the penton base remain unclear and warrant further study. The Hexon protein is a key factor in inducing host production of serotype-specific antibodies (Qiu et al., 2012; Yuan et al., 2009). The results of this study showed that the Hexon antigenic epitopes of the nine HAdV-B strains from Lanzhou City did not exhibit mutations compared to those of the Guangzhou vaccine candidate strain (GenBank: DQ099432.4). This finding suggests that the vaccine candidate strain from Guangzhou, China, demonstrates a theoretical protective potential against the prevalent HAdV-B strain in Lanzhou, based on antigenic epitope conservation. However, its efficacy requires empirical validation through serological cross-neutralization assays to ensure the accuracy and reliability of the results. However, it is worth noting that, compared with the US vaccine strain (GenBank: AY594255.1), the nine HAdV-B strains from Lanzhou City exhibited mutations in all five hexon antigenic epitope regions. These suggest that the US vaccine strain (GenBank: AY594255.1) may provide inadequate immune protection against the currently circulating HAdV-B strains in Lanzhou.

## 5 Conclusion

In summary, this study analyzed the epidemiological and molecular evolutionary characteristics of HAdV in acute respiratory infection cases in Lanzhou City between 2023 and 2025. The results showed that HAdV primarily infects children, with outbreaks occurring throughout the year but primarily concentrated in autumn and winter. Additionally, this study identified amino acid mutations in the functional regions of the major capsid protein of HAdVs. Based on the above findings, to effectively address HAdV epidemics and potential mutations, we propose the following targeted control strategies: strengthen regional surveillance programs by establishing a systematic regional monitoring framework. Development strategies for multivalent, multi-strain vaccines incorporating HAdV-B3 and other known potential epidemic genotypes should be explored. Consideration of antigenic characteristics derived from locally circulating strains, particularly HAdV-B3 isolates from Lanzhou, is essential to ensure that vaccine immunogenicity is suitable for target populations. Vaccine design should incorporate known key antigenic sites to enhance cross-protection against the circulating strains. Through enhanced regional surveillance and forward-looking vaccine development strategies, we can more effectively address HAdV epidemics, provide scientific evidence for public health decision-making, and reduce the disease burden associated with HAdV.

## Data Availability

The original contributions presented in the study are included in the article/supplementary material. Further inquiries can be directed to the corresponding author.
